# Five-year outcomes of chordal replacement therapy for primary mitral regurgitation: The CHAGALL (CHordArt System Study for the Treatment of Mitral ReGurgitAtion due to Leaflet ProLapse or fLail) study

**DOI:** 10.1016/j.xjon.2026.102055

**Published:** 2026-08-13

**Authors:** Alberto Weber, Kestutis Rucinskas, Jodi Akin, Maurizio Taramasso, Michel Zuber, Rolf Vogel, Andrej Podkopajev, Vilius Janusauskas, Diana Zakarkaite, Thomas Bauer, Thierry Carrel

**Affiliations:** a Heart Center Hirslanden, Zurich, Switzerland; b Clinic of Cardiovascular Diseases, Institute of Clinical Medicine, Faculty of Medicine, Vilnius University, Vilnius, Lithuania; c CoreMedic, GmbH, Radolfzell, Germany; d Department for Cardiology, Hospital Solothurn, Solothurn, Switzerland; e Department of Cardiac Surgery, University Hospital, Basel, Switzerland

**Keywords:** mitral valve, regurgitation, artificial chords, automated procedure, transcatheter technique

## Abstract

**Objectives:**

The CHAGALL (CHordArt System Study for the Treatment of Mitral ReGurgitAtion due to Leaflet ProLapse or fLail) study is a prospective, first-in-human early feasibility study evaluating the technical feasibility, the long-term safety, and device durability of a novel chordal replacement system for the treatment of primary degenerative mitral regurgitation.

**Methods:**

Five patients with symptomatic severe primary degenerative mitral regurgitation attributable to isolated central posterior leaflet prolapse or flail underwent surgical implantation of the ChordArt chordal replacement system through a left atrial approach under cardiopulmonary bypass combined with ring annuloplasty. Clinical and transthoracic echocardiographic follow-up was prospectively performed at discharge and through 60 months.

**Results:**

Technical device success was achieved in all patients. Mitral regurgitation was reduced to none or trace immediately after implantation and remained stable throughout the 5-year follow-up. No device-related major adverse events, structural device failure, implant dislodgement, chordal rupture, reoperation, or rehospitalization occurred. Reverse cardiac remodeling was observed, including reduction in left ventricular end-diastolic diameter and left atrial volume, whereas left ventricular function remained preserved during long-term follow-up.

**Conclusions:**

Surgical implantation of the ChordArt chordal replacement system was simple to perform and demonstrated excellent long-term technical durability and sustained clinical performance over 5 years. These findings support further clinical evaluation of this technology and the ongoing development of a fully transcatheter transseptal delivery platform.

**Figure:**
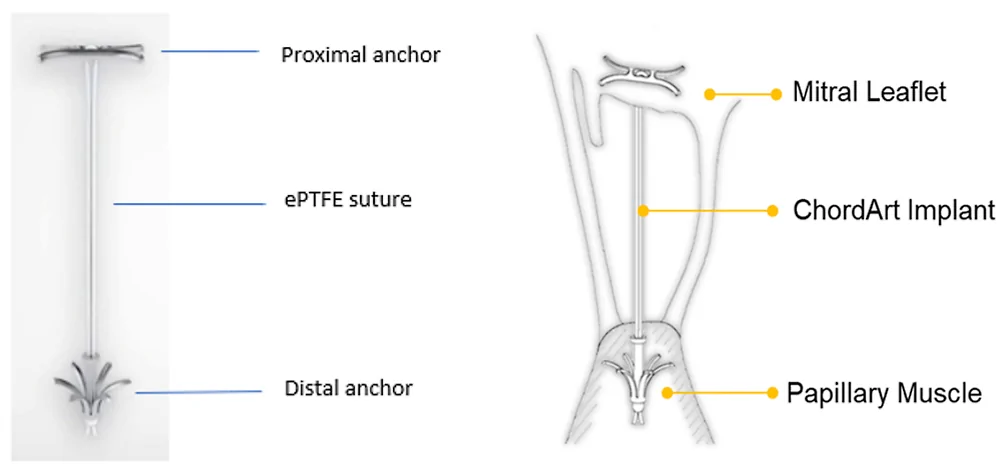
Implant with proximal anchor, the ePTFE suture, and the distal anchor for papillary muscle.

**Central Message**

The automated ChordArt implant was used in 5 patients with isolated posterior leaflet prolapse. They all showed immediate MR reduction and fully stable results up to 5 years.

**Perspective**

This is the first-in-human experience with surgical implantation of artificial chordae using the ChordArt implant with sustained benefits at 5 years. A catheter-based system as an early treatment option and an alternative to surgery or Mitra-Clip for patients at high surgical risk is already underway.

Mitral regurgitation (MR) resulting from structural abnormalities such as leaflet prolapse, flail, chordae tendineae rupture, or anulus dilatation is among the most common valvular heart diseases, with prevalence increasing with age and affecting up to 2% of the general population.^1^ Surgical intervention is required in approximately 5% of individuals older than 75 years and in more than one half of all patients needing treatment, with reported mortality rates of approximately 1.2% at 1 year and survival of 70% to 80% at 5 years, depending on comorbidities.^2^

The ChordArt System (CoreMedic GmbH) is a novel technology developed to enable simple, rapid, and reproducible implantation of artificial mitral chordae. Although initially designed for surgical implantation under direct vision, the technology was conceived as a platform for future minimally invasive and fully transcatheter transseptal procedure. Previous reports demonstrated technical feasibility and favorable short-term outcomes.^3^^,^^4^

The primary objective of the present paper was to evaluate long-term technical feasibility, procedural safety, and device durability over 5 years. Secondary objectives included sustained reduction of MR, clinical status, and echocardiographic evidence of reverse cardiac remodeling.

## Methods

### Study Design and Oversight

The CHAGALL (CHordArt System Study for the Treatment of Mitral ReGurgitAtion due to Leaflet ProLapse or fLail; ClinicalTrials.gov Identifier: NCT03581656) study was a prospective, single-center, single-arm, nonrandomized clinical designed to evaluate the technical feasibility, safety, and efficacy of the ChordArt mitral chord implantation device. This study represents the final 5-year analysis of the first-in-human early feasibility cohort. Clinical and echocardiographic follow-up was performed at baseline, hospital discharge, and at 1, 12, 24, 36, and 60 months after procedure.

Patients were enrolled between April and August 2018 at Vilnius Heart Surgery University Hospital, Vilnius, Lithuania. The study was sponsored by the device manufacturer (CoreMedic GmbH). The protocol was approved by the national regulatory authorities and the Vilnius Regional Biomedical Research Ethics Committee (approval number 158200-18-980-484, approved February 13, 2018). All patients provided written informed consent for publication before participation.

### Patient Selection

Seven patients were screened, but 2 were excluded because intraoperative assessment revealed anatomical characteristics not fulfilling the inclusion criteria, including nonisolated posterior leaflet pathology and insufficient leaflet anatomy for safe device implantation (Figure E1). Five patients aged ≥18 years with symptomatic severe primary degenerative MR attributable to isolated central posterior leaflet prolapse or flail, with left ventricular ejection fraction (LVEF) >25%, and left ventricular end-systolic diameter (LVESD) <55 mm, were enrolled according to the study protocol. All patients were suitable candidates for surgical mitral valve (MV) repair according to the former guideline recommendations available at the time of the study^5^^,^^6^ and were approved by a multidisciplinary Heart Team composed of a cardiac surgeon, an interventional cardiologist, and an echocardiography specialist. Key exclusion criteria were asymptomatic primary degenerative MR; secondary (functional) MR due to annular dilation (leaflet-to-annulus index >1.4); anterior or bileaflet MV prolapse, with or without paracommissural involvement; significant leaflet or annular calcification (anatomical types C or D); severe left ventricular systolic dysfunction (LVEF <25% and/or left ventricular end-diastolic diameter [LVEDD] >6.5 cm); life expectancy <1 year due to noncardiac comorbidities; New York Heart Association functional class IV; concomitant aortic or tricuspid valve disease requiring intervention; previous cardiac surgery; severe pulmonary hypertension (systolic pulmonary artery pressure >70 mm Hg); and renal insufficiency (serum creatinine >200 μmol/L [2.25 mg/dL]). Full eligibility criteria are provided at ClinicalTrials.gov (NCT03581656).

### Study Protocol

All patients underwent both transthoracic echocardiography (TTE) and transesophageal echocardiography (TEE). Anatomical suitability of the MV morphology was assessed by the screening committee. Echocardiographic images were analyzed by an unblinded core laboratory (Academic Core Lab, Ultrasound-based Cardiovascular Imaging, Comprehensive Heart Failure Center) using ImageArena 4 (TomTec Imaging Systems). The MV was evaluated from periprocedural TEE 3-dimensional datasets acquired before intervention, using the 4D MV-Assessment 2 software package (TomTec Imaging Systems). Left ventricular global longitudinal strain (GLS), LVEDD, LVESD, and LVEF were measured using the semiautomated 4D LV-Analysis 3 software.

The severity of MR was quantified using both semiquantitative and quantitative methods and graded as none/trace, mild, moderate, or severe, according to the criteria of the American Society of Echocardiography and the European Society of Cardiology.^7^^,^^8^ TTE was performed at baseline (before the procedure), before hospital discharge, and at 1, 12, 24, 36, and 60 months after ChordArt implantation.

### ChordArt Device and Operative Technique

The ChordArt implant consists of 3 main components: the proximal anchor (a nickel titanium nitinol anchor for leaflet fixation), the artificial chord consisting of a CV-5 expanded polytetrafluoroethylene (Gore-Tex, W. L. Gore & Associates) suture; and the distal anchor (a nickel titanium anchor for papillary muscle fixation) (Figure 1). The distal anchor comprises a 3-piece assembly, including a crown and hub, which are welded to the shaft. The implant is manufactured using metal alloys and expanded polytetrafluoroethylene materials commonly used in cardiovascular devices. Fixed-length devices are available in multiple sizes and are preselected during screening based on echocardiographic and/or magnetic resonance imaging measurements.

**Figure 1:**
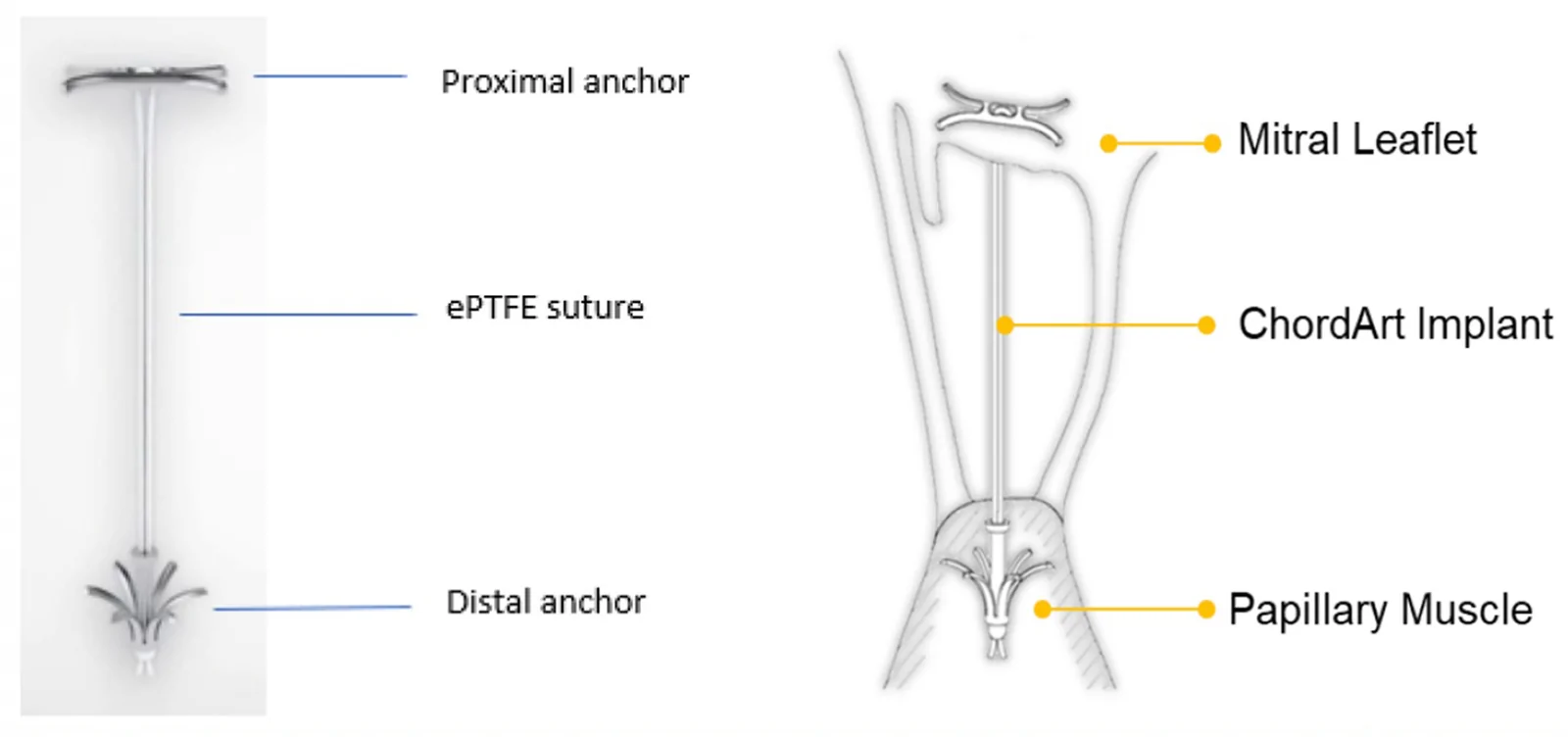
Components of the ChordArt implant. The ChordArt implant consists of 3 main components: the proximal anchor (NiTi anchor for leaflet securement), the chord (ePTFE suture), and the distal anchor (NiTi anchor for papillary muscle attachment). The distal anchor is a 3-piece assembly comprising a crown and hub welded to the shaft. The implant is manufactured using biocompatible metal alloys and ePTFE, materials commonly employed in cardiovascular implants. *NiTi*, Nickel titanium; *ePTFE*, expanded polytetrafluoroethylene.

Implantation of the ChordArt device was performed under cardiopulmonary bypass through a standard sternotomy, as previously described.^4^ Patients were prepared and treated according to standard cardiac surgical protocols. The implant length was selected on the basis of preoperative TEE measurements. After the prolapsing leaflet segment was grasped, the leaflet was punctured approximately 3 to 4 mm from the free edge using a 1.5-mm delivery needle, followed by puncture of the target papillary muscle to deploy the distal anchor. The device was then retrieved, and once the proximal anchor reached the atrial surface of the leaflet, it was released to secure the prolapsing segment. Steps were repeated if multiple chordae were required to restore optimal MV function. All patients underwent thereafter complete ring annuloplasty using a semirigid Séguin annuloplasty ring (Abbott/St Jude Medical) ranging from 30 to 38 mm that was fixed according to standard surgical technique. Procedures were performed by a team of experienced cardiac surgeons and interventional cardiologists.

### Statistical Methods

Given the pilot nature of this study and the small sample size, no formal hypothesis testing was performed. Descriptive statistics were used to summarize study outcomes. Categorical variables are presented as counts and percentages, whereas continuous variables are expressed as mean ± SD (range) or as mean with 95% CIs. All statistical analyses were performed using R software, version 4.0.2 (R Foundation for Statistical Computing).

## Results

Five consecutive male patients (mean age, 63 ± 11 years) with symptomatic severe primary degenerative MR caused by isolated posterior leaflet prolapse underwent successful MV repair with implantation of the ChordArt device. Baseline left ventricular systolic function was preserved (mean LVEF 62 ± 6% by TEE and 61 ± 4% by TTE), and all patients were in New York Heart Association functional class II or III (Table 1). The device was implanted successfully in all patients without need for valve replacement, second cardiopulmonary bypass run or intra-/postoperative systolic anterior motion. Mean cardiopulmonary bypass time was 97 ± 29 minutes, and mean aortic crossclamp time was 69 ± 17 minutes.

**Table 1:** Baseline demographic, clinical, and preoperative echocardiographic characteristics of the study population

Variable	All (n = 5)
Age, y, mean ± SD	63 ± 11
Gender male, n (%)	5 (100)
BMI, kg/m^2^, mean ± SD	27.5 ± 3.8
Hypertension, n (%)	3 (60)
Diabetes, n (%)	0 (0)
Smoking, n (%)	0 (0)
Hyperlipidemia, n (%)	0 (0)
NYHA class II, n (%)	4 (80)
NYHA class III, n (%)	1 (20)
Echocardiographic characteristics, mean ± SD	
LVEF-TEE, %	62 ± 6
LVEF-TTE, %	61 ± 4
LVEDD, mm	61 ± 3
LVESD, mm	41 ± 3
GLS, %	20.62 ± 3.2

Data are expressed as mean ± SD or as number (%). *BMI*, Body mass index; *NYHA*, New York Heart Association functional status; *LVEF*, left ventricle ejection fraction; *TEE*, transesophageal echocardiography; *TTE*, transthoracic echocardiography; *LVEDD*, left end-diastolic diameter; *LVESD*, left ventricular end-systolic diameter; *GLS*, global longitudinal strain.

All patients underwent successful MV repair, with 2 artificial chords used in 4 patients and a single ChordArt artificial implant in 1 patient. (lengths: 14 mm in P2 targeting the anterolateral papillary muscle; 11 mm in P2 targeting the posteromedial papillary muscle).

### Effectiveness Outcomes

MR reduction to none or trace (<1+/4+) was achieved immediately after chordal implantation in all patients and was maintained at the 60-month follow-up (Table 2). No dehiscence, detachment, or dislocation of any ChordArt device was observed.

**Table 2:** Echocardiographic evaluation during the study period

Ultrasound and clinical parameters	Baseline	Discharge	1 mo	12 mo	24 mo	36 mo	48 mo	60 mo
LVEF, %∗	61.8 (54.6-68.9)	52.6 (44.3-60.9)	57.0 (47.6-66.4)	56.8 (47.6-65.9)	62.0 (55.4-68.6)	59.6 (53.2-65.9)	58.8 (51.7-65.9)	57.4 (47.2-67.6)
LVEDD	61.4 (57.4-65.4)	53.8 (49.9-57.7)	51.6 (45.8-57.4)	50.2 (42.9-57.4)	48.2 (43.8-52.6)	51.0 (47.6-54.4)	52.4 (48.3-56.5)	51.6 (44.9-58.3)
LVESD	41.0 (37.8-44.2)	45.4 (40.9-49.8)	41.2 (34.1-48.2)	37.6 (32.9-42.3)	35.2 (29.5-40.8)	38.0 (32.9-43.1)	40.4 (32.9-47.9)	40.2 (29.5-50.9)
GLS, %	20.6 (16.7-24.6)	14.9 (11.3-18.5)	18.5 (15.6-21.3)	19.8 (16.9-22.7)	20.0 (14.9-25.0)	20.0 (14.9-25.0)	16.7 (13.1-20.3)	16.6 (13.4-19.8)
LAV	–	100.8 (70.2-131.4)	72.0 (58.5-85.5)	70.2 (62.4-78.0)	64.0 (54.6-73.4)	64.2 (53.6-74.8)	69.0 (65.6-72.4)	79.8 (57.4-102.2)
MR 4+	5/0	0/5	0/5	0/5	0/5	0/5	0/5	0/5
NYHA II	4/5	2/5	4/5	5/5	5/5	5/5	5/5	5/5
NYHA III	1/5	3/5	1/5	0/5	0/5	0/5	0/5	0/5

Ultrasound parameters at baseline, discharge, 1, 12, 24, 36, 48, and 60 months are shown. *LVEF*, Left ventricle ejection fraction; *LVEDD*, left end-diastolic diameter; *LVESD*, left ventricular end-systolic diameter; *GLS*, global longitudinal strain; *LAV*, left atrial volume; *MR*, mitral regurgitation; *NYHA*, New York Heart Association functional status.

∗ Left ventricle ejection fraction obtained with transthoracic echocardiography.

As expected after successful correction of chronic MR, an early postoperative decline in LVEF (15%-18% relative reduction from baseline) and GLS was observed, reflecting normalization of left ventricular loading condition and increase in afterload rather than deterioration in myocardial function. Both parameters recovered during follow-up and remained stable thereafter. Progressive reductions in LVEDD, LVESD, and left atrial volume were consistent with sustained reverse cardiac remodeling (Figure 2, Table 3). At 1- and 5-year follow-up, LVEF remained stable compared with both baseline and 1-month measurements. Similar temporal trends were observed for GLS.

**Figure 2:**
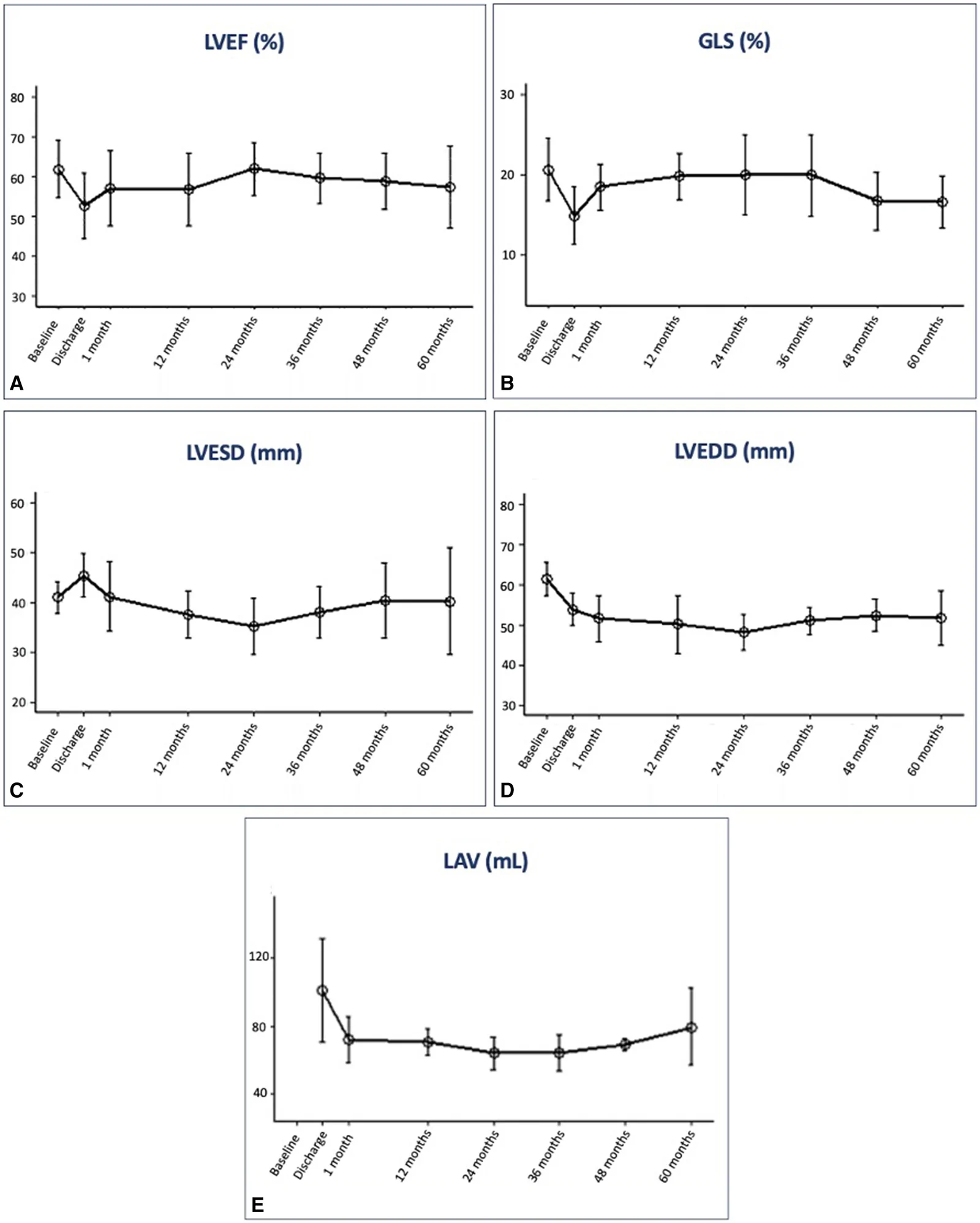
Changes in echocardiographic parameters from baseline to 5-year follow-up. Changes in LVEF (A), GLS (B), LVESD (C), LVEDD (D), and LAV (E) . *LVEF*, Left ventricle ejection fraction; *GLS*, global longitudinal strain; *LVESD*, left ventricle end-systolic diameter; *LVEDD*, left ventricle end-diastolic diameter; *LAV*, left atrial volume.

**Table 3:** Individual echocardiographic and clinical follow-up data through 60 months

Ultrasound and clinical parameters	Alive	MR	LVEF	Leaflet thickening	Leaflet prolapse	GLS abnormality	Pericardial effusion	NYHA functional status	AEs since last visit
60 mo									
Pt 01	Yes	None	47	No	No	No	No	II	PVCs, not device-related
Pt 02	Yes	None	62	No	No	No	No	II	No
Pt 03	Yes	None	68	No	No	No	No	II	No
Pt 04	Yes	None	55	No	No	No	No	II	No
Pt 05	Yes	None	65	No	No	No	No	II	No
48 mo									
Pt 01	Yes	None	49	No	No	No	No	II	No
Pt 02	Yes	None	58	No	No	No	No	II	No
Pt 03	Yes	None	55	No	No	No	No	II	No
Pt 04	Yes	None	60	No	No	No	No	II	No
Pt 05	Yes	None	55	No	No	No	No	II	No
36 mo									
Pt 01	Yes	None	55	No	No	No	No	II	No
Pt 02	Yes	None	55	No	No	No	No	II	No
Pt 03	Yes	None	55	No	No	No	No	II	No
Pt 04	Yes	None	55	No	No	No	No	II	No
Pt 05	Yes	None	55	No	No	No	No	II	No

*MR*, Mitral regurgitation; *LVEF*, left ventricle ejection fraction; *GLS*, global longitudinal strain; *NYHA*, New York Heart Association; *AE*, adverse event; *PVC*, premature ventricular contraction; *Pt*, patient.

### Safety Outcomes

No evidence of implant dislodgement, papillary muscle anchor instability, leaflet anchor dehiscence, chordal or leaflet rupture, structural deterioration, or recurrent leaflet prolapse was observed during the entire 5-year follow-up. No device-related serious adverse events, as defined per protocol, occurred during the procedure or throughout follow-up. Four periprocedural adverse events were recorded, including epigastric pain, clonus after extubation, respiratory infection and paroxysmal atrial fibrillation (successfully treated with cardioversion); all were considered related to the procedure. At 3 months, 1 patient experienced paroxysmal atrial fibrillation that was successfully treated with cardioversion. At 5 years, one patient developed premature ventricular contractions which were managed medically. All adverse events resolved without sequelae, and none were considered to be device-related. Importantly, no patient required reoperation, re-hospitalization nor MV reintervention during the 5-year follow-up period.

## Discussion

Current treatment options for mitral repair include open-heart surgery or more nascent minimally invasive catheter-based therapy, including transcatheter edge-to-edge repair (MitraClip).^9^ Surgical approaches include annuloplasty (ring placement), leaflet repair (eg, repair of prolapsed leaflets with or without resection), replacement of chordae tendineae, and procedures on papillary muscle. Benefits include preservation of the native valve as well as better long-term LVEF and improved survival. Despite favorable long-term outcomes,^10^^,^^11^ surgery is often considered a “watch-and-wait” option mainly because of its invasiveness with prolonged recovery time.

This report presents the longest available clinical follow-up of the first-in-human experience with the ChordArt chordal replacement system. The principal findings are that surgical implantation is technically feasible, the anchoring mechanism remains structurally stable over 5 years, and durable correction of MR can be achieved without device-related major adverse events. Importantly, this study was not designed to demonstrate superiority over conventional MV repair nor to evaluate the independent contribution of artificial chordal replacement in the presence of concomitant annuloplasty. Rather, the main objective was to confirm procedural feasibility, device safety and long-term implant durability. The absence of structural deterioration, anchor dislodgement, recurrent prolapse, or chordal failure after 5 years provides encouraging evidence regarding long-term implant durability and preservation of an optimal MV function. Although the present cohort is small, these observations are particularly relevant because durability remains one of the major concerns associated with novel chordal replacement technologies.

The ultimate goal of this device is to enable a percutaneous procedure, further reducing invasiveness and associated disadvantages of open-heart surgery. Considering the favorable results (safety, effectiveness, and durability) and thanks to the development of a catheter-based transseptal delivery system, a feasibility study of percutaneous ChordArt implantation is currently underway (CHAGALL-TS). This study will have to demonstrate procedural reproducibility and long-term durability with a completely percutaneous approach.

The implications and potential indications for a safe and reproducible catheter-based chordal replacement apply to 2 patient groups: (1) patients with degenerative MR at high surgical risk as the result of advanced age, frailty, and/or comorbidities. These patients are increasingly treated with percutaneous edge-to-edge techniques, which have shown favorable results but are less physiological when the valve function is considered and do not correct concomitant annular dilation In addition they limit the opportunity for later valve repair or replacement (surgical or transcatheter) in case of failure and represents a trade-off between MR reduction and increased valve gradient due to reduced valve area, especially in patients with extensive pathology. (2) Patients with single-segment flail in the early phase of the disease, in whom the annulus is not yet dilated. In this subset, a device that provides physiological repair with a minimal footprint on the valve is appealing. Importantly, unlike edge-to-edge techniques, chordal replacement does not “burn bridges” or limit the possibility of future valve repair or replacement in case of residual or recurrent MR. If proven safe, this early treatment approach could be justified even in younger operable patients because it is minimally invasive and would not preclude successful later repair surgery.

Given the shift from surgical or transapical procedures toward percutaneous and transseptal approaches, there is a trend in device development currently in preclinical testing.^12^, ^13^, ^14^, ^15^ We envision that the expanding toolbox of procedures might expand the population of patients that may be treated earlier in their disease, before significant ventricular deterioration occurs.

### Study Limitations

Several limitations deserve consideration. First, this first-in-human early feasibility study enrolled only 5 carefully selected patients with isolated posterior leaflet prolapse, limiting generalizability. Second, all patients underwent concomitant ring annuloplasty; therefore, the independent contribution of chordal replacement to long-term valve competence cannot be determined. Third, the study was not randomized and lacked a contemporary surgical control group. Accordingly, these findings should be considered hypothesis-generating and require confirmation in larger prospective multicenter studies. Subsequent development efforts focused primarily on the fully transcatheter iteration of the technology, reflecting strategic prioritization of resources and the anticipated future demand for chordal replacement therapy.

## Conclusions

This study represents the first-in-human experience with direct-view, antegrade surgical implantation of artificial chordae using the ChordArt implant to correct primary MR caused by posterior mitral leaflet prolapse or flail in the presence of an annuloplasty ring. ChordArt implantation proved feasible, with no device-related complications reported and sustained clinical and echocardiographic benefits at 5 years. These findings support the development of a catheter-based, transseptal delivery system as an early treatment option or for patients at high surgical risk as alternative toi surgery and Mitra-Clip. Nevertheless, larger studies are needed to confirm the short- and long-term efficacy and safety of the device, while a feasibility trial of the percutaneous transcatheter approach is currently underway.

### Webcast

You can watch a Webcast of this AATS meeting presentation by going to: https://www.aats.org/resources/five-year-outcome-of-the-chaga-12533.

**Figure:**
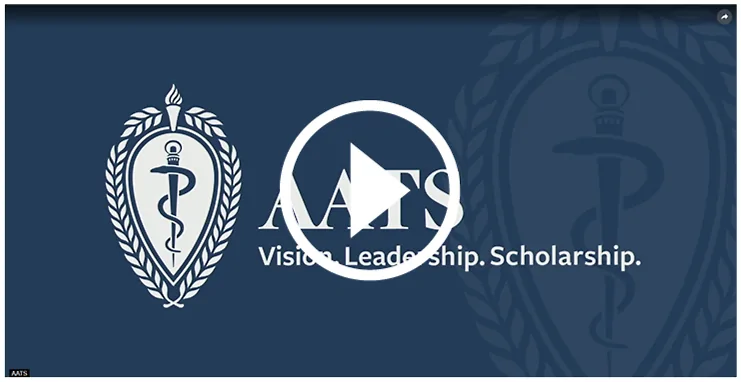

## Conflict of Interest Statement

Dr Weber reported a shareholder relationship with CoreMedic GmbH. Dr Akin reported Chairwoman of the Board and Chief Clinical Officer positions at CoreMedic GmbH. Dr Bauer reported a Chief Technology Officer position and a shareholder relationship with CoreMedic GmbH. Dr Taramasso reported a consultant contract with CoreMedic GmbH. Dr Zuber reported a consultant contract with CoreMedic GmbH. Dr Vogel reported a shareholder relationship with CoreMedic GmbH. Dr Bauer reported a shareholder relationship with CoreMedic GmbH. Dr Carrel reported a shareholder relationship with CoreMedic GmbH. All other authors reported no conflicts of interest with CoreMedic GmbH.

The *Journal* policy requires editors and reviewers to disclose conflicts of interest and to decline handling or reviewing manuscripts for which they may have a conflict of interest. The editors and reviewers of this article have no conflicts of interest.

## Figures and Tables

**Figure E1:**
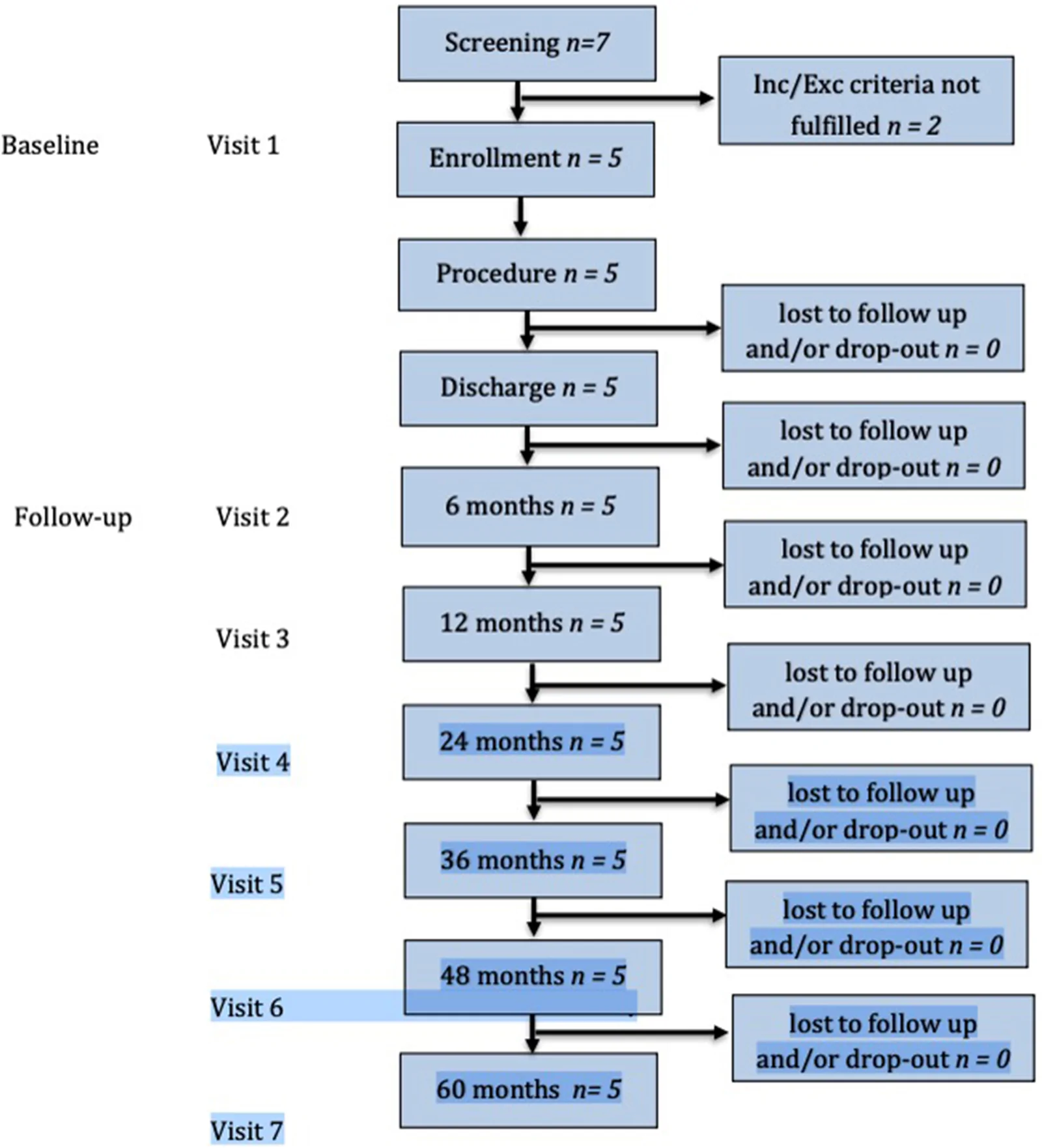
Algorithm of the study, including number of patients screened, enrolled, operated, discharged and followed for up to 5 years.
